# Prevalence and factors associated with inappropriate use of treadmill exercise stress test for coronary artery disease: a cross-sectional study

**DOI:** 10.1186/s12872-015-0048-7

**Published:** 2015-06-16

**Authors:** Antônio M. L. Silva, Anderson C. Armstrong, Fernando J. C. Silveira, Marcelo D. Cavalcanti, Fernando M. F. França, Luis C. L. Correia

**Affiliations:** School of Medicine and Public Health, Salvador, Brazil; University of São Francisco Valley –UNIVASF, Petrolina, Brazil

**Keywords:** Exercise testing, Treadmill test, Coronary artery disease, Appropriateness criteria

## Abstract

**Background:**

In some countries, the public health system has less availability when compared to the population covered by health insurance. In addition, inappropriate referrals for treadmill exercise stress test increase spending and lead to unnecessary interventions. We aim to determine the prevalence and characteristics of inappropriate referrals for treadmill exercise stress tests in the assessment of coronary artery disease (CAD), considering public and private health systems scenarios.

**Methods:**

A cross-sectional design was used to describe the frequency of inappropriate use of exercise testing in the diagnosis of CAD and to determine its predictors. We consecutively enrolled 191 patients from two outpatient facilities in Northeast Brazil. For inclusion, the exercise testing should be referred for the assessment of CAD. We performed logistic regression models to identify independent predictors of inappropriate use.

**Results:**

Treadmill exercise stress tests were rated as inappropriate in 150 (78 %) patients. The majority of patients had low or very low pre-test probability of CAD. Presence of hypertension, diabetes and dyslipidemia were more frequent in the appropriate than inappropriate indications (71 %, 19 % and 29 % *versus* 43 %, 8 % and 16 %, respectively). Tests performed both at the public and private system showed high prevalence of inappropriate examinations, higher in the latter (57 % *versus* 87 %, P < 0.001). The private health system was the major independent predictor of inappropriate referral, consistent in all regression models (when adjusting for clinical variables, OR = 4.3; P < 0.001).

**Conclusion:**

The vast majority of treadmill exercise stress test referrals in the assessment of CAD were inappropriate. The availability of the method and not the estimate probability of CAD appear to be the underlying condition for a treadmill test referral.

## Background

Evidence has failed to show that an aggressive screening for coronary artery disease (CAD) may improve cardiovascular goals in primary prevention. Also, searches for ischemic response in asymptomatic CAD patients are not related to favorable outcomes [1]. The treadmill exercise stress test appears to be particularly vulnerable to overuse in clinical settings, due to its wide availability and affordability. The inappropriate use of the method, however, may result in overdiagnosis and overtreatment of coronary artery disease [2, 3].

Developing countries in which private and public health systems coexist have limited funding for health. In developed countries that also have universal health care systems such as France and Germany, investment in health was about five times higher [4]. The inappropriate use of procedures contributes to the waste of resources that could enhance benefits to the population to be assisted, if they were utilized rationally. Even in developed countries, the growing expenditure on health is a major concern. At least 20 % of health spending in the US represent unnecessary expenses, where the inappropriate use of diagnostic evaluations contribute to this number [5].

Studies that assess inappropriate use of exercise stress tests and their impact remain scarce. This study aimed to assess the frequency and the determinants of an inappropriate use of treadmill exercise stress tests for CAD in outpatient public and private facilities.

## Methods

### Study population

Between November 2012 and April 2013, we consecutively enrolled patients aged ≥18 years, who had been referred to treadmill exercise stress tests for evaluation of CAD from two outpatient facilities in Northeast Brazil. All patients underwent a clinical assessment by experienced cardiologists to evaluate symptoms and risk factors. The pretest probability for CAD was determined based on Diamond-Forrester algorithm, which takes into account chest pain characteristics, age, and sex [6]. The assessment of cardiovascular risk (CV) was defined according to risk prediction of the World Health Organization (WHO), based on sex, age, blood pressure, presence of diabetes and smoking status. Participants were then classified as low if CV risk was estimated to be  < 10 % in ten years, intermediate if between 10 % and 20 %, and high cardiovascular risk if above 20 % [7].

We calculated a sample size of 189 participants, based on an accuracy of ± 7 % and on an estimate of the inappropriate referrals in 60 % of the treadmill tests. The project was approved by the Ethics Committee in Research of Bahia Foundation for Science Development, Brazil. The term informed consent was obtained from all participants.

### Definition of inappropriate use of treadmill exercise stress test

There is no specific guideline on appropriate use criteria for treadmill exercise stress test. However based on the evidence of screening in asymptomatic and assessment in those stable patients with CAD, but also the probabilistic concept of using the method according to the probability of coronary artery disease, we defined the inappropriate diagnostic evaluation parameters. Treadmill exercise stress tests were then rated as inappropriate if they presented one of the following two criteria: (1) patients asymptomatic for CAD; (2) symptomatic patients with low or high pretest probability of CAD. Treadmill exercise stress test in individuals above 70 years of age with chest pain was rated as appropriate, as well as those patients symptomatic with known CAD. Individuals younger than 30 years were classified as the likelihood of CAD according to age group 30-39 years in the criteria of Diamond-Forrester.

### Statistical analysis

The prevalence of inappropriate use was described by proportion and its accuracy estimated by a confidence interval at 95 %. In univariate analysis, variables were compared between inappropriate versus appropriate tests by the chi-square test for categorical variables and Student *t* test for numerical variables.

To identify independent predictors of inappropriate use, variables with P <0.10 in the univariate analysis were included in multivariate logistic regression models using the backwards technique. Three logistic regression models were used to represent the associating parameters derived from the univariate analysis. These models are also compared to clinical thinking in the diagnostic evaluation request. The model 1 is adjusted for clinical variables (hypertension, dyslipidemia and diabetes); model 2 for the number of risk factors and the model 3 is associated with cardiovascular risk. These associations were also described by odds ratios and 95 % CI. Variables that remained in the final model with P <0.05 were defined as independent predictors. The calibration of the models was assessed by testing Hosmer-Lemeshow. We used SPSS software for statistical analysis (version 17.0, SPSS Inc., Chicago, USA).

## Results

We consecutively enrolled 221 participants referred to treadmill exercise stress tests. Thirty patients had requests for evaluation of arrhythmia, systemic blood pressure or functional assessment and were excluded from the study. The research or DAC assessment was the indication for treadmill exercise stress test in 191 subjects. The mean age was 48 ± 14 years, 66 % were female and half of the patients were asymptomatic. There was a predominance of subjects with pre-test probability for coronary heart disease low or very low (29 % and 44 %, respectively) and low cardiovascular risk (80 %), according to the WHO risk prediction. Private health systems diagnostic evaluations represented 71 % of the sample. Other characteristics are described in Table 1.

**Table 1:** Personal Characteristics of Study Sample

Parameters	Values
n	191
Age (years)	48 ± 14
Male	85 (44 %)
Symptom of chest pain^a^	
Asymptomatic	97 (50.8 %)
Nonanginal	80 (41.9 %)
Atypical angina	9 (4.7 %)
Typical angina	5 (2.6 %)
Pre-test probability of CAD	
Very low	84 (44 %)
Low	55 (28.8 %)
Intermediate	29 (15.2 %)
High	1 (0.5 %)
Not applicable^b^	22 (11.5 %)
Hypertension	93 (49 %)
Diabetes	20 (10 %)
Dyslipidemia	36 (19 %)
Obesity	48 (25 %)
Family history of CAD	41 (21 %)
Smoker	12 (6.3 %)
Known CAD	12 (6.3 %)
Previous myocardial infarction	8 (4.2 %)
Revascularization	11 (5.8 %)
Cardiovascular risk^c^	
Low	151 (80 %)
Intermediate	20 (10 %)
High	19 (10 %)
Tests of public health system	56 (29 %)

^a^Symptom in the anamnesis; ^b^Age ≥ 70 years or known CAD; ^c^Cardiovascular Risk Estimation in 10 years, according to the WHO risk prediction (Low: <10 %, Intermediate: between 10 and 20 %, High: ≥ 20 %)

### Prevalence and associated variables of inappropriate treadmill exercise stress tests

For 150 (78 %) participants that underwent treadmill exercise stress tests, the diagnostic evaluations were rated as inappropriate. But both private and public health systems showed high frequencies of inappropriate treadmill exercise stress tests, with higher rates in the private health system (87 % and 57 % respectively; P < 0.001).

Participants undergoing inappropriate treadmill exercise stress tests were significantly younger than those who performed appropriate tests (45 ± 13 years vs 60 ± 12 years, P <0.001). Risk factors such as hypertension, diabetes and dyslipidemia were less frequent when an inappropriate referral was identified, reflecting mostly low cardiovascular risk profiles. Additional clinical characteristics were similar between participants with appropriate and inappropriate treadmill exercise stress tests (Table 2).

**Table 2:** Patient Characteristics According to Treadmill Exercise Stress Test Appropriateness Criteria

Parameter	Appropriate	Inappropriate	*P* Value
	(*n* = 41)	(*n* = 150)	
Male	21 (51 %)	64 (43 %)	0.32
Age	60 ± 12	45 ± 13	<0.001
Clinical conditions			
Diabetes	8 (19 %)	12 (8,0.%)	0.03
Dyslipidemia	12 (29 %)	24 (16 %)	0.05
Hypertension	29 (71 %)	64 (43 %)	0.001
Obesity	12 (29 %)	36 (24 %)	0.49
Previous infarction	2 (4.9 %)	6 (4.0 %)	0.68
known CAD	4 (9.8 %)	8 (5.4 %)	0.29
Revascularization	4 (9.8 %)	7 (4.7 %)	0.25
Smoker	1 (2.4 %)	11 (7.3 %)	0.25
Family history of CAD	10 (24 %)	31 (21 %)	0.60
Risk factors^a^			0.03
No risk factor	6 (14,6 %)	51 (34 %)	
1 or 2 risk factors	25 (61 %)	79 (52,7 %)	
>2 risk factors	10 (24,4 %)	20 (13,3 %)	
Cardiovascular risk^b^			0.001
Low	24 (58.5 %)	127 (85.2 %)	
Intermediate	10 (24.4 %)	10 (6.7 %)	
High	7 (17.1 %)	12 (8.1 %)	
Public health system	24 (58 %)	32 (22 %)	<0.001

^a^Number of risk factors (hypertension, diabetes, dyslipidemia, obesity, family history of CAD, smoking)

^b^Cardiovascular Risk Estimation in 10 years, according to the WHO risk prediction (Low: <10 %, Intermediate: between 10 and 20 %, High: ≥ 20 %)

### Independent predictors of inappropriate treadmill exercise stress tests

Three logistic regression models were used to represent the associating parameters derived from the univariate analysis. Age was not included in the models because this variable was part of the calculation of the pre-test probability (used in the definition of inappropriate use of treadmill exercise stress test). In model 1, among the clinical variables only hypertension was shown to be inversely associated with inappropriate use of the treadmill exercise stress test. In Model 2 and Model 3 revealed no significant association between the number of risk factors or cardiovascular risk with inappropriate indications for treadmill exercise stress test (Table 3). In second moment, we adjusted the model 1 for age, only in order to verify that the inverse association of hypertension with inappropriate testing was mediated by these patients being younger. In fact, hypertension lost statistical significance in this model, while additional network of health remained strongly associated (when adjusting for age, OR = 3.5; P = 0.003). Therefore, patient being in the private health care system was the only important significant covariate in the models, with OR = 4.3 (P < 0.001), according to the model adjusted for clinical conditions. The P value of the Hosmer-Lemeshow test for all models was > 0.05 indicating a good calibration.

**Table 3:** Predictors in Multivariable Logistic Regression for Inappropriate Use of Treadmill Exercise Stress Test for Coronary Artery Disease

	Model 1		Model 2		Model 3	
Predictors	OR (95 % IC)	*P* Value	OR (95 % IC)	*P* Value	OR (95 % IC)	*P* Value
Private health system	4.3 (2.0-9.1)	<0.001	5.1 (2.4-10.6)	<0.001	4.7 (2.2-10.3)	<0.001
Clinical conditions	0.49 (0.17-1.5)	0.200	Not selected		Not selected	
Diabetes		0.505				
	0.74 (0.30-1.8)	0.013				
Dyslipidemia		0.186				
Hypertension	0.37 (0.17-0.81)					
Obesity						
	0.54 (0.22-1.3)					
Risk factors (reference: without risk factor)	Not selected		3.2 (0.98-10.6)	0.054	Not selected	
			1.4 (0.56-3.7)			
1 or 2 risk						
				0.450		
factors						
>2 risk						
factors						
Cardiovascular risk (reference low risk)	Not selected		Not selected		1.9 (0.63-5.8)	0.247
Intermediate						0.161
High					0.37 (0.09-1.5)	

*OR: Odds Ratio*

## Discussion

In a developing country, we describe the prevalence of inappropriate use of treadmill exercise stress tests for the diagnosis of CAD in public and private health system facilities. The vast majority of diagnostic evaluations were rated as inappropriate. Being in the private health system increased over 4.3 folds the odds of being referred for an inappropriate treadmill exercise stress test. Moreover, patients’ clinical characteristics appear to have had little impact on treadmill referrals.

We showed a remarkably high proportion of inappropriate treadmill exercise stress test in our population, which seems to be in line with other areas of the world. An Italian study evaluated 960 referrals for non-invasive cardiac testing, finding that only a third of the requests were considered appropriate. Moreover, exercise testing had only 27 % of appropriate referrals, showing the worst results among non-invasive cardiac tests [8]. Studies evaluating appropriateness criteria in the use of treadmill exercise stress tests for diagnosis of obstructive CAD are still sparse in the literature. However, evidences show that asymptomatic individuals do not benefit from active screening for obstructive CAD [9–12]. Additionally, mandatory myocardial stress testing in patients with known CAD did not reduce major CV outcomes [1, 13, 14]. Similarly, individuals in both low and high pre-test probability for obstructive CAD do not benefit from treadmill test screening, as results may not substantially affect the likelihood of disease. Therefore, the main utility of the exercise test in the diagnosis of obstructive CAD lies in symptomatic patients with intermediate pretest probability.

Although we found a high frequency of inappropriate treadmill exercise stress tests among participants from the public health system, inappropriate diagnostic evaluations were even more frequent in patients from the private health care system.

In Brazil two healthcare systems coexist: the public and the private. The evident imbalance between the funding expenses for private and public health in a developing country as Brazil leads to an unequal health policy. In December 2013 there were 2109 offices specialized in the public system, while in the private network had 8937 specialized consultation rooms [15]. It reflects the higher availability of health services in the private healthcare system, compared to the scarce resources in the public system. Although covering only 24 % of the population, about 54 % of the spent in the Brazilian healthcare in 2011 was in the private system. The public health system covers the majority of the Brazilian population, investing around 512 dollars per person per year [4, 16]. Our multivariable models showed that only being from the private health system was an independent predictor of inappropriate use of treadmill exercise stress test. Remarkably, neither the cardiovascular risk nor the presence of comorbidities tested in our logistic regression models remained significant after adjusting for health system. Apparently, the main variable when deciding to use a treadmill exercise stress test in the study was the availability of the method. The prevalence of heart disease in the age group between 35 and 49 years of age is approximately 3.7 % in Brazil [17]. Indeed, characteristics of the inappropriate sample (mean age of 45 and low cardiovascular risk profiles) do not seem representative of a usual treadmill referral population. These findings are an emblem of health consumerism from people who have health insurance and can get (unnecessary) testing.

Irrational use of diagnostic complementary methods directly impacts healthcare spending. The cost of cardiovascular disease is significant in Brazil, with an expense of about 1.74 % of Gross National Product [17]. In 2012, 497,242 treadmill exercise stress tests have been performed in the Brazilian public health system, with a cost of more than $ 7 million US dollars. With our results in mind, nearly 243 thousand exams could be considered inappropriate across Brazil, representing an unnecessary expense of more than $ 3 million dollars in 2012 [18]. In addition to that, inappropriate treadmill exercise stress tests may lead to unnecessary additional procedures, generating potential harm to patients and also increasing costs in health systems. A Canadian study showed that 42 % of 2,718 patients with suspected CAD had a normal cardiac catheterization, although the majority had a previous positive noninvasive stress test [19]. In a US study with 397,954 patients, most elective catheterizations showed no obstructive coronary disease, although over 80 % of the patients underwent a previous noninvasive test [20]. Furthermore, a normal treadmill exercise stress test may negatively affect primary prevention strategies, as the wrong perception of absent coronary disease may prevent susceptible individuals from reducing their modifiable risk factors and overall risk profile.

There are limitations regarding the generalization of our study. In fact, it is a small sample and the results may represent the local reality of a center in Northeastern Brazil. Further research assessing different populations in other health centers may reinforce our findings and broaden the discussion of this important topic.

## Conclusion

We found a high prevalence of inappropriate treadmill exercise stress test referrals for the diagnosis of CAD. This finding was consistent in both private and public health systems, but higher in the private system. Absence of comorbidities such as hypertension, diabetes and dyslipidemia, as well as judgment to estimate cardiovascular risk were not associated with inappropriate indication for use of treadmill exercise test in the diagnosis of obstructive CAD. In fact, the private health care system was the only predictor of inappropriate indications. It is likely that the greater availability of complementary method in that system, favoring its inappropriate use. Our findings may provide insights that favor the judicious use of medical resources even when largely available, focusing on the best evidence and the appropriate use of the complementary method diagnostic.
